# Income-Related Gaps in Early Child Cognitive Development: Why Are They Larger in the United States Than in the United Kingdom, Australia, and Canada?

**DOI:** 10.1007/s13524-018-0738-8

**Published:** 2018-11-28

**Authors:** Bruce Bradbury, Jane Waldfogel, Elizabeth Washbrook

**Affiliations:** 1 Social Policy Research Centre, University of New South Wales, Sydney, NSW 2052 Australia; 2 Columbia University School of Social Work, 1255 Amsterdam Avenue, New York, NY 10027 USA; 3 School of Education, University of Bristol, Bristol, BS8 1JA UK

**Keywords:** Child development, School readiness, Parental income, Cross-national, Social mobility

## Abstract

Previous research has documented significantly larger income-related gaps in children’s early cognitive development in the United States than in the United Kingdom, Canada, and Australia. In this study, we investigate the extent to which this is a result of a more unequal income distribution in the United States. We show that although incomes are more unequal in the United States than elsewhere, a given difference in real income is associated with larger gaps in child test scores there than in the three other countries. In particular, high-income families in the United States appear to translate the same amount of financial resources into greater cognitive advantages relative to the middle-income group than those in the other countries studied. We compare inequalities in other kinds of family characteristics and show that higher income levels are disproportionately concentrated among families with advantageous demographic characteristics in the United States. Our results underline the fact that the same degree of income inequality can translate into different disparities in child development, depending on the distribution of other family resources.

**Electronic supplementary material:**

The online version of this article (10.1007/s13524-018-0738-8) contains supplementary material, which is available to authorized users.

## Introduction

It has long been established in the intergenerational mobility literature that social mobility is lower in the United States than in many advanced nations (Corak 2006; Solon 2002). Recent work has sought to understand when in the life course the impact of socioeconomic status (SES) on children’s life chances in the United States begins to diverge from that in other countries (Blanden et al. 2014; Ermisch et al. 2012a). Research by Bradbury et al. (2012, 2015a) has shown that as early as age 5, significantly stronger SES gradients exist in children’s early cognitive development in the United States compared with the United Kingdom, Canada, and Australia. This finding applies whether SES is measured using parental education (Bradbury et al. 2015a) or quintiles of household income (Bradbury et al. 2012). Although both measures of SES have their advantages, the income-based definition arguably better reflects a country’s overall policy environment because it depends not only on parental human capital in terms of educational levels but also on the interaction of this human capital with the labor market and the tax and transfer system. Previous research comparing income-related gaps in children’s development across countries has used a relative definition by dividing families into groups using equal-sized within-country quintile group definitions. Hence, it is not clear whether the greater inequality in children’s test scores found in the United States is a consequence solely of a greater level of income inequality in that country. Put simply, it is possible that the rich are richer and the poor are poorer in the United States than elsewhere and that greater inequalities appear in early childhood development in a relative sense only because we are not comparing “like with like” across countries. Alternatively, a given degree of income inequality may matter more in the United States because other contextual factors play a more reinforcing role.

In this study, we test these interpretations by documenting the gaps in children’s early language and literacy skills in four nationally representative cohort studies when groups are defined according to *absolute* income thresholds. Specifically, we calculate the quintile boundaries (in real equivalized family income in dollars) for a representative U.S. sample of 5-year-olds; we then convert these dollar boundaries to the other countries’ currencies and use them to divide representative samples of children from the United Kingdom, Australia, and Canada into groups with incomes similar to those in the United States. Gaps in mean test scores among the different income groups then give a measure of inequality that compares children with the same levels of financial resources across countries. The distribution of income in the United States is more unequal than in the other countries. Nonetheless, gaps in early cognitive development remain significantly larger in the United States even when income groups are defined in this absolute manner. We show that a given absolute disparity in financial resources is not magnified into the same gap in children’s school readiness in the three other countries as it is in the United States.

What might account for greater U.S. gaps in school readiness if not financial resources themselves? A number of factors can potentially explain why the same incomes translate into different environments for children in different countries. For example, a mother with low skills working long hours may generate the same income as a mother with high skills working shorter hours, but we would expect both the quantity and quality of maternal time inputs into child well-being to be higher in the latter situation. A greater proportion of income generated from other sources, such as a partner’s earnings or state transfers, may also free up maternal resources more in some countries than others. Cost and availability of quality childcare will also be important in terms of the out-of-home environments that children with a given level of income have access to, as well as how much money is left to the family after childcare expenditure. Similarly, access to health care would have both direct effects on child development and indirect effects via the effect on family budgets. We compare income-related inequalities in a range of other family characteristics across countries to see which, if any, have the potential to account for the weaker association between income and school readiness found in the non-U.S. countries. Our results reveal that families with incomes that would place them in the upper quintiles of the U.S. income distribution are relatively less advantaged in the United Kingdom, Australia, and Canada than they are in the United States in terms of family structure, maternal age, parental nativity, maternal nonwork time, and access to childcare. The greater concentration of nonfinancial resources among those with higher monetary resources in the United States, therefore, may explain why income seems to matter more for children’s school readiness in the United States.

## Background and Related Literature

A large body of work has compared the income-related gradients in offspring’s income or earnings across different countries, states, and cohorts (e.g., Bjorklund and Jäntti 2009; Blanden et al. 2004; Chetty et al. 2014; Corak 2006; Lee and Solon 2009; Solon 2002). This literature has established that substantial differences in intergenerational income mobility exist across societies with, for example, the United States typically displaying greater income persistence and less mobility across generations than a number of Western European countries and, in particular, the Nordic countries (Corak 2013; Jäntti et al. 2006). Quite naturally, these descriptive patterns spur inquiry into the underlying reasons for the differences. Child development scholars have long been interested in the links between parental economic resources and children’s development (Bradley and Corwyn 2002; Conger et al. 1992; Duncan and Brooks-Gunn 1997; McLoyd 1998), but it was perhaps the formalization of James Heckman’s dynamic model of lifecycle human capital accumulation (Cunha et al. 2006) that encouraged social mobility researchers to look back for the origins of intergenerational income persistence in childhood. A good deal of evidence is now available on how income-related gradients evolve across different domains of human development (cognitive, socioemotional, and health), different life course stages (from infancy through to early adulthood), and different times and places (Bailey and Dynarski 2011; Berger et al. 2009; Blanden et al. 2007; Case et al. 2002; Fletcher and Wolfe 2016; Martinson and Reichman 2016; Reardon 2011; Waldfogel and Washbrook 2011; Washbrook et al. 2014).

The early childhood period attracts particular attention because of evidence that plasticity in certain developmental domains declines rapidly as children age (Almond and Currie 2011; Knudsen et al. 2006). However, until recently, cross-national comparison of SES-related gaps early in childhood has been hampered by lack of data. International large-scale assessments, such as the Programme for International Student Assessment, have long enabled comparisons at older ages, but large-scale nationally representative direct measures of young children’s cognitive and socioemotional skills, accompanied by detailed measures of parental SES, have been rare. Bradbury et al. (2012, 2015a) addressed this gap by drawing together and harmonizing data from independent nationally representative cohort studies from the United States, the United Kingdom, Australia, and Canada.

Bradbury et al. (2012) presented the gaps in mean standardized test scores at age 5 among different SES groups for cohorts born between 2000 and 2004. They operationalized SES in two ways: by parental education level and by within-country quintile of equivalized parental income. They showed that regardless of whether SES is measured by education or income, the U.S. gaps in test scores between SES groups at age 5 are significantly larger than in all three other countries, suggesting that low U.S. levels of social mobility have their origins early in life, prior to children’s school entry. Bradbury et al. (2015a, b) provided a more detailed study that investigated disparities in children’s environments as well as children’s test scores over a range of ages, across the four countries, for cohorts born between 1991 and 2002. That study focused almost entirely on gaps in test scores by parental education among these cohorts, providing only brief results for gaps in age 5 test scores by within-country income quintile groups in a technical appendix (Bradbury et al. 2015b:46). Notably, the cross-country differences across the studies were remarkably stable, confirming that the finding of greater U.S. test score gaps is not dependent on the use of particular cohorts, data sets, or measures of SES.

Parental education has advantages as a measure of SES in terms of its simplicity, accuracy of measurement, stability over the child’s life course, and correlation with social and cultural as well as economic capital (Ermisch et al. 2012b). Previous work has focused on the relationship between parental education and other family resources across countries (McLanahan and Jacobsen 2015). However, as the long history of work on intergenerational income persistence demonstrates, the specific link between parental monetary resources and children’s outcomes is of distinct interest. This is particularly true from a public policy perspective: parental incomes of the current generation are relatively malleable via taxes, transfers, and labor market regulations, whereas levels of parental education are likely much harder to shift, at least in the short term.

The gaps in test scores by income quintile group presented in Bradbury et al. (2012, 2015b) addressed this interest directly. Compared with a single correlational income-outcome statistic, the widely used income quintile group approach permits nonlinear relationships and provides an accessible summary of cross-group comparisons (Blanden and Machin 2010; Carneiro and Heckman 2003; Magnuson et al. 2012; OECD 2013; Waldfogel and Washbrook 2011).

When used in comparative work, the income quintile group approach imposes an ordinal interpretation of socioeconomic stratification: the richest one-fifth in different countries are assumed to be equally advantaged by virtue of their rank, even if they have quite different levels of financial resources at their command. This relative approach is appropriate if one wishes to remove the effects of national differences in income inequality and isolate differences in the degree of *association* between income and the outcome, as has been the case in the vast majority of studies in the intergenerational mobility literature (Black and Devereux 2011; Solon 1999). However, the approach has drawbacks from a more policy-focused perspective that seeks to understand the role of material resources in generating gaps in children’s development in different countries. It is not clear from the results in Bradbury et al. (2012, 2015b), for example, whether income matters more for children’s life chances in the United States or whether between-group parental income disparities are simply greater in the United States (or both). Dividing parents into groups with the same absolute levels of income, rather than ranking positions cross-nationally, helps to clarify this question. If fixed differences in income translate into the same gaps in school readiness in other countries as in the United States, then this points to wider income inequality as the source of the larger U.S. gaps between within-country income quintile groups. If, however, U.S. gaps in school readiness between equal-income groups remain larger than those in other countries, we must look to disparities in other nonmonetary factors to explain the differences in gaps in school readiness across groups based on rank.

In neither case can we argue that gaps in school readiness between children in rich families and those in poor families are entirely caused by income differences. Too many other important parental characteristics, such as education and family composition, are strongly correlated with income. But the combined effect is nonetheless important. Compellingly demonstrating this idea in the context of changes over time, McLanahan (2004) argued that the strengthening of the association between socioeconomic resources and demographic characteristics over time in the United States is leading to “diverging destinies.” In this article, we tackle a similar issue, focusing on the comparison of countries at a single point in time rather than of cohorts within a single country.

The nonmonetary factors that we consider are informed by an ecological model of child development (Bronfenbrenner 1994). This approach posits that the proximal determinants of child development—the interactions and lived environments experienced by the child—are shaped by more distal factors at the level of family, community, and society. For example, Wadsworth and Ahlkvist (2015) argued that economic inequality is the root cause of the differences in parenting behavior that ultimately account for a large portion of the achievement gap. Financial resources are one distal factor, and they matter for children not just in terms of the child investment in goods and services (such as good neighborhoods) that parents are able to purchase (the *child investment perspective*) but also in terms of how they affect family functioning and stability (the *family stress perspective*) (see, e.g., Guo and Harris 2000; Linver et al. 2002). Other distal factors also matter for the proximal environment, and the characteristics that we examine in this study have all been long-discussed as influences on child development. Specifically, we consider the composition of income groups in terms of maternal education (Carneiro et al. 2013; Magnuson 2007); family structure (Ermisch and Francesconi 2001; McLanahan and Sandefur 1994); maternal age, and specifically teen motherhood (Conger et al. 1984; Geronimus et al. 1994); parental nativity (Crosnoe and Turley 2011; Washbrook et al. 2012); maternal employment patterns (Lucas-Thompson et al. 2010; Waldfogel et al. 2002); and exposure to preschool education and center-based care (Duncan and Magnuson 2013; Yoshikawa et al. 2013). The strength of the association between each of these factors and income will be the outcome of a complex set of interactions between the demographic composition of a society, the economic structure of the labor market, and the public policy environment (Corak 2013; Nolan et al. 2011; Solon 2004). Different degrees of association may therefore arise for many reasons, and our presumption is only that, all else being equal, societies in which a given factor is more evenly distributed across income groups will tend to have smaller (or at least not larger) gaps in test scores than societies in which the factor is more stratified by income.

## Data and Methods

Our data are taken from four nationally representative cohort studies, details of which are summarized in Table 1. All the studies collected measures of children’s language and literacy skills around age 5 via direct assessment, as well as rich data on family background and children’s environments via parent self-report. All the studies surveyed children around ages 5 and 11 and at least one time point in between. The United States, United Kingdom, and Australian surveys sampled a single birth cohort, and we use these samples in their entirety. The Canadian survey contains data on multiple cohorts of children born between 1983 and 2008; for comparability with the other samples, we restrict our analysis of these data to children from the Original Cohort born between 1991 and 1994. Extensive details of the studies, the analysis samples used here, and variable construction can be found in Bradbury et al. (2015b). Briefly, we use data from the longitudinal samples of children present in the surveys between the age 5 and age 11 waves of data collection. In all analyses, we use the age 11 longitudinal survey weights provided by the survey administrators, along with all available data on survey design features, to adjust for attrition and nonrandom sampling. Although we focus here on child test scores from the age 5 waves only, the longitudinal sampling rule is appropriate because it allows for direct comparability with the results in Bradbury et al. (2015b), and (as discussed later) improves the accuracy of income measurement by allowing us to average reports of family income from multiple waves.

**Table 1:** Data sources

	United States	United Kingdom	Australia	Canada
Survey	Early Childhood Longitudinal Study, Kindergarten Cohort (ECLS-K)	Millennium Cohort Study (MCS)	Longitudinal Study of Australian Children Kindergarten Cohort (LSAC-K)	National Longitudinal Study of Children and Youth (NLSCY)
Cohort Birthdates	1992–1993	9/2000–1/2002	3/1999–2/2000	1/1991–12/1994
Achieved Sample at Baseline	19,170	18,818	4,980	8,605
Analysis Sample Size	8,370	11,762	3,900	4,298
Mean Child Age at Cognitive Assessment	5.7 years (Fall K)	5.2 years	4.9 years	4.9 years
Early Literacy Measurement Instrument	ECLS-K Reading Test	BAS Naming Vocabulary Test	PPVT-III	PPVT-R
Study Information	Tourangeau et al. (2009)	Hansen (2014)	AIFS (2013)	Statistics Canada (2007)

### Age 5 Test Scores

The age 5 test scores are measures of children’s early language and literacy. Of the domains of development assessed in the cohort studies, these measures are the most useful for cross-national comparisons because of the similarity of their content and because, as direct assessments, they are arguably more objective than measures based on parent or teacher reports. Although the test instruments differed across the country surveys, all tests were administered by interviewers using an easel and required children to use only pointing or verbal responses to complete the tasks: children were not asked to write anything or to explain their reasoning in any of the assessments. All the tests were adaptive and routed children to different items based on their previous responses, using item response theory (IRT) methods to derive a measure of the child’s underlying ability (known as the theta score in the ECLS-K).

The Canadian and Australian survey instruments were both versions of the Peabody Picture Vocabulary test. The Australian LSAC-K employed a shortened version of the PPVT-III (Dunn et al. 1997; Rothman 2005), while the Canadian NLSCY used an older version, the PPVT-R, and its French language equivalent, the EVIP (Dunn and Dunn 1981; Dunn et al. 1993). UK children were assessed using the British Ability Scales (BAS) Naming Vocabulary test (Elliott et al. 1997), which is similar to the PPVT but tests expressive rather than receptive vocabulary. The U.S. test was the ECLS-K Fall Reading test (Tourangeau et al. 2009). This assessment included questions designed to measure receptive vocabulary but also two domains not covered explicitly by the assessments in the other three countries: basic literacy skills (print familiarity, letter recognition, beginning and ending sounds, rhyming sounds, and word recognition) and comprehension (listening comprehension and words in context). The inclusion of items related to letters and print is a distinct feature of the ECLS-K data, but it is unlikely to substantially affect estimates of the test score gaps.^1^ The ability scores provided in the official data files were first purged of variation related to within-sample differences in children’s age at testing by taking the residuals from a regression of each test score on a cubic polynomial of child’s age in months at assessment. These residuals were then standardized to mean zero, unit variance *z* scores using the appropriate longitudinal survey weight.

### Family Income Measures

We use the measures of average family pretax post-transfer equivalized income derived by Bradbury et al. (2015b).^2^ In all surveys, this measure is the mean from three reports of household income when the cohort child was roughly 5, 7, and 11 years of age. This procedure is important because single-period estimates of family income are likely to contain high degrees of measurement error that will tend to attenuate estimates of the association with test scores. Averaging income reports over multiple periods better approximates the concept of permanent income and can make a dramatic difference to our understanding of gaps in test scores (Rothstein and Wozny 2013). Income data were collected somewhat differently in each of the surveys, and the decision to harmonize to a gross (i.e., pretax and post-transfer) measure of income was judged to involve imposition of the fewest assumptions (for further details, see Bradbury et al. 2015b). At each wave, family income was converted to constant 2011 prices using the relevant national price index and then converted to U.S. dollars using the OECD purchasing power parity (PPP) index for actual individual consumption for 2011. Estimates were equivalized for household size (*N*) by multiplying by the scaling factor 2 / √*N* (so that the numbers presented correspond to the reference income of a family of four) and were then averaged across the three waves. Income quintile boundaries were calculated for the U.S. sample using the appropriate longitudinal weight, and then these dollar boundaries were used to split all four country samples into five comparable income groups.

Table 2 shows the income thresholds used to define the U.S. income quintile groups and the distribution of children in the other three countries across groups defined by these dollar thresholds. While 20 % of the U.S. population of 5-year-olds were in households with an income of below $27,000 per year (equivalized to a family of four), a far smaller fraction fell below this threshold in the other three countries. Children in the second and third groups, with incomes between $27,000 and $65,000, were overrepresented in the other countries relative to the United States. At the higher end of the distribution, the income distribution is virtually identical in the United Kingdom and the United States, but notably smaller fractions of children with family incomes over $96,000 are found in Australia and particularly in Canada than in the United States.

**Table 2:** Percentage of sample in income groups defined by U.S. income quintile boundaries

	Q1: <$27K	Q2: $27K–$44K	Q3: $44K–$65K	Q4: $65K–$96K	Q5: >$96K
United States	20.0	20.0	20.0	20.0	20.0
United Kingdom	13.0	23.9	22.5	20.1	20.6
Australia	10.1	21.1	27.4	24.4	17.1
Canada	12.1	25.3	30.3	22.0	10.3

*Note*: Incomes are gross (pretax but post-transfer), equalized and normalized to a family of four, and expressed in 2011 U.S. dollars.

For groups defined in this way to be truly comparable, we require the assumption that the distribution of incomes *within* these U.S.-defined groups is the same across countries. The average group incomes shown in Table 3 suggest that this holds well for the lower four groups, but those in the top income group—above the $96,000 threshold—may still be richer, on average, in the United States than in the other countries. This difference should be considered when comparing the top income group with the others.

**Table 3:** Mean incomes (thousands of 2011 U.S. dollars) in groups defined by U.S. income quintile boundaries

	USQ1: <$27K	USQ2: $27K–$44K	USQ3: $44K–$65K	USQ4: $65K–$96K	USQ5: >$96K
United States	19.4	35.2	54.2	79.4	159.6
United Kingdom	20.9	35.3	54.4	78.5	122.6
Australia	20.4	35.5	53.8	77.9	139.0
Canada	21.1	36.1	54.2	77.9	132.3

*Note:* Incomes are gross (pretax but post-transfer), equalized and normalized to a family of four, and expressed in thousands of 2011 U.S. dollars.

### Measures of Nonmonetary Resources Available to Children

We categorize maternal education levels into three levels, harmonizing to the U.S. definitions: high school or less, some college, and college degree (bachelor’s degree) or more. Family structure is captured by an indicator for whether the child resides with both biological parents at age 5. A dummy variable for teen parenthood indicates whether the child’s mother was under 20 at the time of his or her birth. Parental nativity is captured by an indicator for whether any parent coresiding with the child at age 5 was born in another country. Maternal employment status is measured by the mother’s usual hours of work as reported at the age 5 interview, and we additionally distinguish between a dummy variable for any participation in paid work at that time point and hours of work conditional on participation. Exposure to early childhood education is represented by an indicator for whether the child attended any kind of preschool, nursery, day care center, pre-kindergarten, or center-based program (such as Head Start) in the year prior to formal schooling (for full details of variable coding, see Bradbury et al. 2015b).

### Estimation Methods

Our use of standardized scores to measure school readiness within each country means that we are able to compare only group differences, rather than absolute levels, across countries. For logical consistency, therefore, our descriptive approach must also compare gaps in inputs across countries. Average *levels* of resources available to children, such as parental educational attainment and preschool attendance, may differ between countries and therefore may influence the average test scores of all children there. What is relevant for understanding gaps in test scores, however, is how resources in the higher- and lower-income groups compare within country with those available to families in the middle reference group.

We characterize all inequalities, in both early test scores and nonmonetary family resources, in terms of differences in group means relative to the group with middle incomes defined using the U.S. income quintile boundaries (i.e., families with incomes of $44,000–$65,000; Q3). Specifically, *X*_*i*,*c*_ is the characteristic of interest of child *i* in country *c*, and *Q*_*i*,*c*_ is a categorical variable for the income group to which the family belongs, taking values from *q* = 1 (the lowest-income group, less than $27,000) to *q* = 5 (the highest-income group, $96,000 or more). For each country *c*, we present estimates of four gaps:

$$ {D}_{c,q}=E\left({X}_{i,c}|{Q}_{i,c}=q\right)-E\left({X}_{i,c}|{Q}_{i,c}=3\right)\kern2.5em q=1,2,4,5 $$

*D*_*c*,1_, therefore, is an estimate of the difference in means between the lowest-income group and the middle-income group: the Q1–Q3 gap. Negative values indicate a lower mean among the specified income group relative to the reference group of middle-income children. The choice of Q3 as the reference category allows us to investigate differences at the lower and upper ends of the income distribution in a consistent way. Occasionally, we also refer to the overall summary measure of the Q5–Q1 gap, *G*_*c*_ = *D*_*c*,5_ – *D*_*c*,1_, which gives the difference in means between the highest and lowest income groups in country *c*.

The sample means and their standard errors are calculated for each income quintile group using the microdata from each country, taking account of relevant weights and survey design features. Approximate standard errors of the differences are calculated assuming independence of the samples in each quintile group. Because our ultimate interest is in cross-national comparisons, we also calculate a set of double differences that measure the difference between a particular gap in the United States, *D*_*US*,*q*_, and each of the three comparator countries: *DD*_*c*,*q*_ = *D*_*US*,*q*_ – *D*_*c*,*q*_,* c* = *UK*, *AU*, *CA*.

The standard errors of the estimates of *D*_*US*,*q*_ and *D*_*c*,*q*_ from independent samples can be combined to test the significance of *DD*_*c*,*q*_—that is, whether a particular gap is significantly different in the United States than in another country.

## Results

### Inequalities in Early Test Scores

**Fig. 1:**
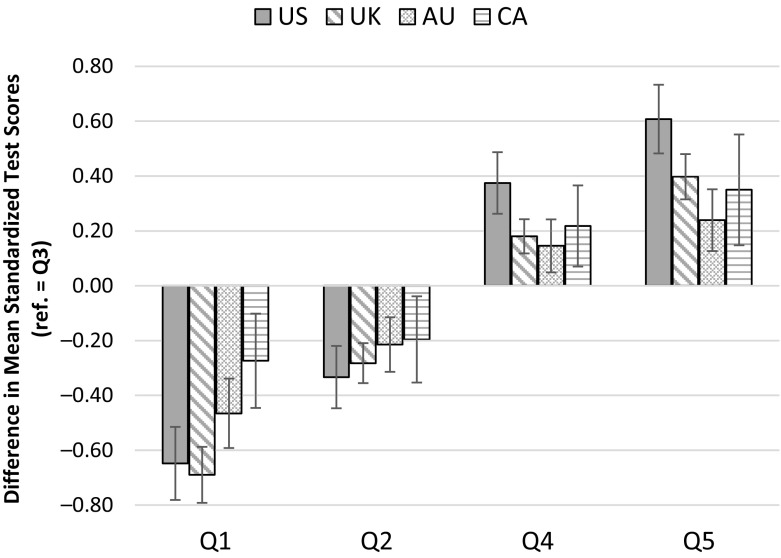
Within-country gaps in mean early literacy test scores (reference category = Q3). Error bars are 95 % confidence intervals. Q1 to Q5 refer to income groups defined by the quintile boundaries of the U.S. distribution from lowest to highest; that is, Q1 is the group with income less than $27,000, and Q5 is the group with income greater than $96,000. The chart plots the gap in mean standardized test scores between the specified group and the middle Q3 group (families with incomes of $44,000–$65,000 equivalized, in 2011 U.S. dollars). Estimates are calculated from the underlying microdata separately for each country. See Tables 1 and 2 for details of the source samples.

Table 4 provides details of the income-related gaps in standardized literacy test scores in each country and also the significance of the differences between the gaps in the United States and those in the three other countries. Figure 1 gives a graphical presentation of the estimates in Table 4.

**Table 4:** Gaps in mean early literacy test scores (reference category = Q3): Within countries and in comparison with the United States

	Q1–Q3 (*D*_*c*,1_)	Q2–Q3 (*D*_*c*,2_)	Q4–Q3 (*D*_*c*,4_)	Q5–Q3 (*D*_*c*,5_)	Overall Gap:Q5–Q1 (*G*_*c*_)
Gaps in Test Scores Within Country					
United States	–0.65	–0.33	0.37	0.61	1.26
United Kingdom	–0.69	–0.28	0.18	0.40	1.09
Australia	–0.47	–0.21	0.15	0.24	0.70
Canada	–0.27	–0.20	0.22	0.35	0.62
Difference in Gaps in Test Scores Between the United States and Other Countries (*D*_*US*,*q*_ – *D*_*c*,*q*_)					(*G*_*US*_ – *G*_*c*_)
United Kingdom	0.04	–0.05	0.19	0.21	0.17
	(0.09)	(0.07)	(0.07)**	(0.08)**	(0.09)
Australia	–0.18	–0.12	0.23	0.37	0.55
	(0.09)	(0.08)	(0.08)**	(0.09)**	(0.10)**
Canada	–0.37	–0.14	0.16	0.26	0.63
	(0.11)**	(0.10)	(0.09)	(0.12)*	(0.13)**

*Notes:* Q1 to Q5 refer to income groups defined by the quintile boundaries of the U.S. income distribution from lowest to highest; that is, Q1 is the group with income less than $27,000, and Q5 is the group with income greater than $96,000. Q3 (families with incomes of $44,000–$65,000) is the reference group in all but the final column. Estimates are calculated from the underlying microdata separately for each country *c*. All scores are standardized. Standard errors are shown in parentheses. See Tables 1 and 2 for details of the source samples.

*p < .05; **p < .01

Looking to the estimates at the bottom right of Table 4 (or the sum of the bars on the far left and the far right in Fig. 1), the overall Q5–Q1 gap in test scores is larger in the United States than in all the other three countries, although not significantly so in the case of the United Kingdom. The literacy scores of children in homes with incomes less than $27,000 per year are markedly more similar to those of children with incomes over $96,000 per year in Australia and Canada than in the United States, by an order of more than one-half of a standard deviation (SD).

The most striking differences between the United States and the other countries are seen in the gaps at the higher end of the income distribution. The bottom panel of Table 4 shows that the gap in test scores between the highest and middle-income groups (the Q5–Q3 gap) is significantly smaller than in the United States by 0.21 SD in the United Kingdom, by 0.26 SD in Canada, and by 0.37 SD in Australia (differences that amount to, respectively, 34 %, 43 %, and 61 % of the 0.61 SD U.S. Q5–Q3 gap). These results indicate that higher-income groups in the United States are able to translate their financial resources into greater cognitive advantages relative to the middle-income group than in any of the other three countries. Or, put another way, children in the United States seem to be more adversely affected by being in the $44,000 to $65,000 income group, rather than a higher-income group, than do children in other countries. This also holds for the Q4–Q3 comparison (significantly so in the case of the comparison with the United Kingdom and Australia), so it cannot be driven solely by higher levels of income among the top income group in the United States.

Gaps in test scores between middle- and lower-income children tend to be more similar across countries. For example, there are no significant cross-country differences in the Q2–Q3 gaps: the gaps between the middle group and those just below it in the $27,000–$44,000 income group are in the range of one-third to one-fifth of a standard deviation in all four countries and are not statistically distinguishable. Only Canada has a significantly smaller Q1–Q3 gap in test scores than in the United States—between children in the lowest income group (<$27,000) and the middle. This difference, however, is very large, at less than one-half the size in Canada as in the United States.

These results are important because they show that the larger gaps in test scores between income groups in the United States, when defined by relative incomes documented in previous work, cannot simply be attributed to the more unequal income distribution in the United States. Compared with the gaps presented in Bradbury et al. (2015b:46), which used an identical sample and data but divided the non-U.S. countries into within-country income quintile groups, the switch to absolute income boundaries does tend to widen the non-U.S. test score gaps, as we would expect. For example, the UK Q5–Q1 gap in test scores becomes insignificantly different from the U.S. Q5–Q1 gap in test scores when the income boundaries are harmonized, and the Canadian Q5–Q3 gap in test scores increases from 0.30 to 0.35 SD when the threshold for being considered high income is raised to $96,000. However, it is clear from Fig. 1 that a given dollar difference in family income predicts a noticeably larger gap in child test scores in the United States than in a number of peer countries, particularly at the higher end of the income distribution.

### Income Inequalities in Other Family Resources

How is it that some countries appear able to limit the consequences of income inequality for early childhood development? The results in the previous section suggest that nonmonetary resources may be more evenly distributed across income groups in the non-U.S. countries and that these equalizing factors should be most pronounced when comparing middle- and higher-income groups.

**Fig. 2:**
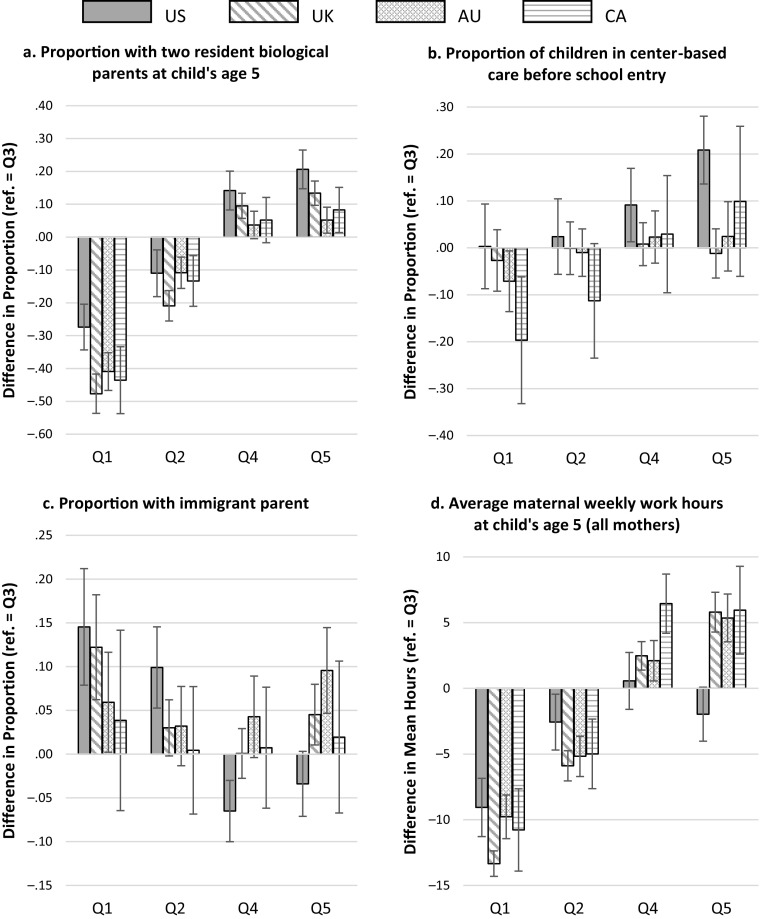
Within-country gaps in family characteristics (reference category = Q3). Each panel plots the gaps in a different variable. Error bars are 95 % confidence intervals. Q1 to Q5 refer to income groups defined by the quintile boundaries of the U.S. distribution from lowest to highest; that is, Q1 is the group with income less than $27,000 and Q5 is the group with income greater than $96,000. Gaps are the differences between the mean or proportion in the group and the middle (Q3) group (families with incomes of $44,000–$65,000). Estimates are calculated from the underlying microdata separately for each country. See Tables 1 and 2 for details of the source samples.

**Fig. 3:**
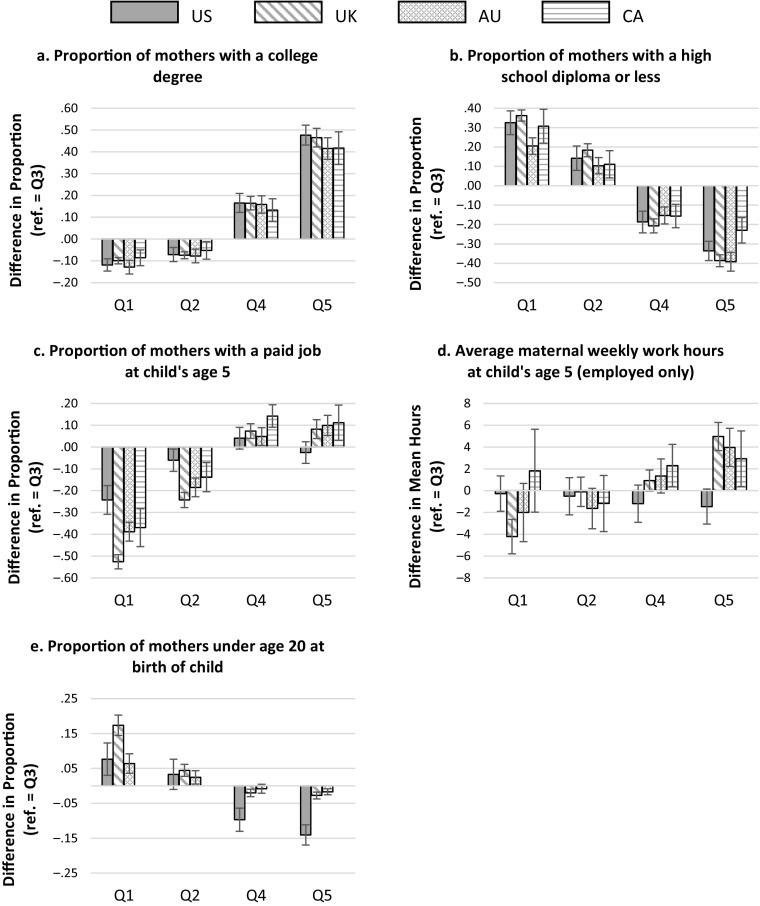
Within-country gaps in additional family characteristics (reference category = Q3). Canadian numbers for teenage motherhood suppressed due to small cell sizes. Each panel plots the gaps in a different variable. Error bars are 95 % confidence intervals. Q1 to Q5 refer to income groups defined by the quintile boundaries of the U.S. distribution from lowest to highest; that is, Q1 is the group with income less than $27,000, and Q5 is the group with income greater than $96.000. Gaps are the differences between the mean or proportion in the group and the middle Q3 group. Estimates are calculated from the underlying microdata separately for each country. See Tables 1 and 2 for details of the source samples.

Table 5 describes how other important influences on early school readiness vary across countries for the Q3 middle-income reference group. These provide the baseline levels used in the calculation of the gaps across income groups shown subsequently in Figs. 2 and 3.

**Table 5:** Characteristics of families with incomes of $44,000–$65,000 (Q3) in the four countries

	United States	United Kingdom	Australia	Canada
Mothers With a High School Diploma or Less (proportion)	.46	.51	.59	.33
Mothers With a College Degree (proportion)	.13	.10	.22	.12
Two Resident Biological Parents at Age 5 (proportion)	.66	.85	.92	.86
Mothers Under Age 20 at Birth of Child (proportion)	.15	.03	.02	––
Immigrant Parent (proportion)	.17	.12	.31	.21
Average Maternal Weekly Work Hours at Age 5 (all mothers)	27.2	16.5	15.7	22.9
Mothers With a Paid Job at Age 5 (proportion)	.74	.69	.66	.74
Average Maternal Weekly Work Hours at Age 5 (employed only)	36.8	24.1	23.8	31.0
Attendance at Center-Based Care in the Year Prior to School Entry (proportion)	.62	.93	.95	.60

*Notes:* See Tables 1 and 2 for details of the source samples. Canadian numbers for teenage motherhood are suppressed because of small cell sizes.

Table 5 reveals that despite variation in the characteristics and conditions of middle-income families, there is no country in which this section of the population stands out as advantaged or disadvantaged compared with its counterparts in other countries.

In terms of demographic characteristics, mothers with all levels of education are represented in the Q3 ($44,000–$65,000) income group in each of the four countries. The proportion with low education is noticeably lower in Canada than elsewhere, and the proportion with a college degree is noticeably higher in Australia. Compared with their counterparts in other countries, fewer middle-income children in the United States resided with both biological parents at age 5, and more were born to teen mothers. The proportion of middle-income children living with an immigrant parent varies from 12 % in the United Kingdom to 31 % in Australia, with the North American groups falling in the middle of this range.

Turning to maternal employment patterns and exposure to preschool in the Q3 (middle-income) families, there is a clear difference between the North American countries on one hand and the United Kingdom and Australia on the other. Household income in these families is more dependent on maternal earnings in the United States and Canada because mothers are more likely to work there and particularly because employed mothers work considerably longer hours than in the United Kingdom and Australia (although working part-time does appear more common in Canada than the United States). Contrary to what this pattern might lead us to expect, (at least some) attendance at center-based childcare in the year prior to formal schooling was almost universal among the middle-income group in the United Kingdom and Australia, two countries with universal preschool systems; in the mid-late 1990s, however, only around 60 % of children attended some center-based care in the United States and Canada, countries with higher maternal employment levels but largely private systems of childcare.

The numbers documented in Table 5 show some variation across countries in the circumstances experienced by children with the same moderate level of pretax financial resources. For our purposes, however, what is of most interest is the extent to which the characteristics of children with lower and higher family incomes differ from their middle-income peers, and whether these differences mirror the differences in gaps in school readiness.

Figure 2 plots the gaps across income groups in selected family characteristics across the four countries, with additional characteristics examined in Fig. 3. For each country, the gaps represent the difference between the mean or proportion for a given family factor in the lower- or higher-income groups and the value in the middle-income ($44,000–$65,000) group. Larger gaps reflect a stronger association between levels of income and the family characteristic across countries. We would expect the characteristics shown in the top panels of Fig. 2 (relating to family structure and early education) to be positively associated with children’s test scores, and hence positive gaps in these panels reflect more advantageous circumstances. The characteristics in the bottom panels (relating to parental nativity and maternal employment hours) are theoretically negatively related to school readiness, all else equal, and so the reverse is the case here: positive gaps indicate less advantageous circumstances. Details of all the underlying estimates and significance tests of differences are provided in the online appendix.

We turn first to the characteristics of families in the two higher-income groups: those with incomes between $65,000 and $96,000 (Q4), and those with incomes in excess of $96,000 (Q5), represented by the right-hand sets of bars in each panel in Fig. 2. As shown in Fig. 1, gaps in school readiness in both these groups relative to the middle-income group are considerably larger in the United States than in all the other three countries.

Figure 2 shows that higher-income children in the United States have greater advantages (relative to their middle-income peers) than those in other countries in all the selected dimensions. Higher-income children in the United States are the most concentrated in intact families (i.e., with both biological parents coresident at age 5) compared with their middle-income peers (shown by the larger positive gaps in panel a). In addition, higher-income children in the United States experience by far the greatest positive difference in preschool attendance relative to middle-income children (see the significantly positive U.S. gaps in panel b). Higher-income children in the United Kingdom and Australia are not significantly more likely than middle-income children to attend center-based care in the year prior to school entry, a consequence of virtually universal coverage in those countries. Income-related disparities in preschool attendance did exist among higher-income groups in Canada at this time, but these are imprecisely estimated and markedly smaller than in the United States. At the same time, higher-income U.S. children are less likely to have an immigrant parent than middle-income children, the only country in which this is the case (see the negative gaps in panel c). Supplemental analyses (Fig. 3) also show that the difference in teen parenthood rates between the middle- and the top-income groups is sharpest in the United States.

With regard to maternal employment patterns, the United States is the only country in which higher incomes come at virtually no cost in terms of greater maternal work hours. Panel d of Fig. 2 shows that the Q4 and Q5 gaps in maternal employment hours are not significantly different from 0 in the United States, in contrast to positive gaps in the other three countries. Outside the United States, the benefits of higher incomes may be tempered by the fact that mothers in these households work more than those in middle-income households, so that children potentially experience less time with their mothers in the home. Mothers in the United States with the highest (family) income are unique in that they appear to devote *less* time to employment than their middle-income counterparts, reinforcing rather than offsetting the inequalities in both income and participation in center-based care. Supplemental analyses show that uniquely, U.S. mothers in Q5 differ little from Q3 mothers in both employment participation and hours of work conditional on participation, with the point estimates suggesting a negative differential in both (Fig. 3). In the other three countries, both participation rates and hours conditional on employment are systematically higher in the higher-income groups than in the middle group, a mechanism that potentially offsets the benefits of higher income. Interestingly, the cross-country differences in inequalities at the top end of the income distribution shown in Fig. 2 are not underpinned by strong differences in the distribution of maternal education. Supplementary analysis provided in Fig. 3 shows an extremely similar association between maternal education (whether captured by attainment of a college degree or failure to progress beyond high school) and income group in all four countries.

In summary, these patterns highlight that in a number of respects, the middle-income group in the United States is more *disadvantaged* relative to children in well-off families than elsewhere. In the other countries, the middle group is less differentiated from the top in terms of demographic characteristics (such as family structure, maternal age, and parental nativity) and in terms of access of early childhood education. In addition, middle-income mothers in the United States must devote more time to employment in order to avoid slipping below an income of $44,000 per year.

These same figures also show how families in the middle-income groups compare with those lower down the income distribution. Recall from Table 4 that the test score gaps between lower- and middle-income groups (i.e., Q1–Q3 and Q2–Q3) in the United States were not significantly different from those in the United Kingdom or Australia, but that children in the lowest group (with incomes of less than $27,000) were faring significantly better in Canada. At this end of the distribution, the position of U.S. children is considerably more complex. In some respects, lower-income children in the United States are less differentiated from their middle-income counterparts than elsewhere. The Q1–Q3 disparities in family structure and preschool attendance are actually smaller in the United States than in any other country (as shown by the smaller negative, or even positive, U.S. gaps in panels a and b of Fig. 2). Disparities in maternal education and rates of teen parenthood between Q1 and Q3 (shown in Fig. 3) are similar in the United States to those in the other countries and in some respects are smaller than those in the United Kingdom. The relatively disadvantaged character of the U.S. middle-income group means that in some respects, their children enjoy only marginally higher nonmonetary resources than the poor, whereas families with moderate incomes in the other countries are more positively differentiated.

However, low-income children in the United States do appear relatively worse off in some ways. Low-income U.S. children were disproportionately likely to be born to an immigrant parent (as shown by the largest positive gaps in panel c, Fig. 2). In contrast, the immigrant difference between Q3 and Q1 is the smallest of the four countries in Canada and not significantly different from 0. Similar to the pattern for middle- and higher-income mothers, low-income mothers in the United States enjoy the least relief from paid employment relative to those higher up the income scale. The small magnitude of the negative U.S. gaps in average hours in panel d (Fig. 2) reflect particularly small differences in the employment rates of Q1 and Q3 mothers compared with those in other countries. Lower-income mothers in the United States, therefore, work nearly as much as those in families with incomes of $44,000 to $65,000 and are less likely to experience the social resources associated with being native-born, disadvantages that are more muted in the other countries. Any drawbacks for children associated with these factors may be counterbalanced, however, by rates of single and teen parenthood and of the use of center-based childcare that are relatively similar to those of the middle-income group.

### The Role of Taxes

Our measure of income takes into account transfers provided to families, including cash benefits, in-kind benefits, and tax credits. However, because of data limitations, we are not able to adjust incomes for taxes paid. Perhaps the stronger association between income and children’s school readiness in the United States reflects the fact that higher-income families are able to retain a higher proportion of their income than in the other three countries. To address this possibility, we look to an external data source—the Luxembourg Income Study (LIS)—for information on how tax rates for families with children vary across countries and gross income levels. We use these data to calculate the average tax rates in each income group as the mean ratio of a household’s total income tax and social insurance payments to total gross household incomes (including transfer payments)^3^ and use these estimates to explore the impact of the progressivity of the tax system.

Table 6 shows the average tax rates in each income group across countries, with greater progressivity of the tax system indicated by larger gaps in average tax rates between the Q3 reference group and the other income groups. The results suggest that larger gaps in school readiness in the United States between higher-income groups and those in the middle (the Q5–Q3 and Q4–Q3 gaps in school readiness) cannot be attributed to a less-progressive tax structure at the top of the income distribution in the United States. In fact, the proportion of income paid in tax rises more sharply (or equally sharply) as incomes increase above the median in the United States than in the other countries. This finding reflects the fact that the middle-income reference group is taxed relatively lightly in the United States compared with other countries, as shown by the average tax rates in the central Q3 column. Comparatively low U.S. tax rates are less marked higher up the income distribution: the highest income group pays an additional 15 % of income in tax in the United States (a 29 % rate compared with a 14 % rate for the middle group), whereas the rise in Canada, for example, is only 10 percentage points (from a 23 % to a 33 % rate). These patterns do not support the conjecture that the higher-income groups are able to retain more of their gross incomes in the United States relative to the middle group and thus extend their children’s comparative advantage more via this mechanism. In contrast, the tax structure at the bottom of the United States income distribution is the least progressive of all the four countries. Compared with those in Q3, those in the lower-income groups in the United States see the smallest reduction in tax rate of all four countries (implying that the gap in disposable income between Q1 and Q3 is largest in the United States). This could potentially explain why the gaps in the school readiness between the bottom and the middle in the United States are not smaller than elsewhere, despite the smaller disparities in single parenthood and early education participation. It is notable that for the lowest Q1 group, the most progressive tax system is found in Canada, where the tax rate for Q1 families is 17 percentage points lower than for the middle Q3 group.

**Table 6:** Average tax rates in groups defined by U.S. income quintile boundaries

	Q1: <$27K	Q2: $27K–$44K	Q3: $44K–$65K	Q4: $65K–$96K	Q5: >$96K
Average Tax Rate (%)					
United States	5	10	14	19	29
United Kingdom	3	11	18	22	23
Australia	0	7	14	19	26
Canada	6	17	23	26	33
Gap Relative to Q3 (percentage points)					
United States	–9	–4	ref.	5	15
United Kingdom	–15	–7	ref.	4	5
Australia	–14	–7	ref.	5	12
Canada	–17	–6	ref.	3	10

*Notes:* Tax rates are the mean percentage of total gross household income (including transfer payments) that is paid in income tax and social insurance payments by households in each income group, calculated from the LIS data. Gaps are expressed in percentage points.

## Conclusions

This study has shown that the larger income-related gaps in child development in the United States documented in previous work using country-specific income groups cannot simply be attributed to the more unequal income distribution in the United States. Our work using income groups defined in absolute terms, using the U.S. income distribution, suggests that higher-income parents in the United States translate a given difference in household income into a greater advantage in terms of children’s school readiness than similarly affluent parents in the United Kingdom, Australia, and Canada. The evidence presented here suggests that this could be due to the relatively high degree of nonmonetary advantages experienced by children in the higher-income groups in the United States, relative to those in the middle-income group, in a number of domains. As noted previously, a broad literature has linked these domains to children’s development through a variety of different mechanisms. First, higher-income children in the United States are disproportionately likely to live with both biological parents and to have mothers who are able to limit the time they devote to paid work, potentially leading to benefits for children via higher levels of nurturing, stimulating parental interactions, and better parental psychological well-being. Second, higher-income children in the United States are disproportionately likely to have only native-born parents, which may confer benefits in terms of parental networks and social support, ability to access services, and children’s language development. Third, higher-income children in the United States are disproportionately likely to attend a center-based preschool, where they may receive additional cognitive stimulation and preparation for the classroom environment. Middle- and high-income families in the other countries are less differentiated in terms of all these characteristics, patterns that sit alongside significantly smaller gaps in children’s test scores between these groups than in the United States.

In contrast, a given income difference at the bottom of the income distribution is associated with similar test score gaps among U.S., UK, and Australian children, with the low-income effect on test scores seemingly smaller only in Canada. The similarity in gaps in test scores between middle-income children and their low-income peers across several countries, however, disguises some important differences in the degree of income-related gaps in different nonmonetary resources. Middle-income families in the United States are relatively similar to their low-income counterparts in having high rates of teen parenthood and single parenthood and low rates of early education; in the other countries, middle-income families are more advantaged in these respects, leading us to expect *greater* test score gaps in the other countries than in the United States. However, low-income children in the other countries are potentially compensated by the fact that their mothers devote relatively less time to paid work than their middle-income counterparts. This may work to narrow the gaps more than in the United States, where low-income mothers’ employment participation is relatively high. Less progressivity in the tax system at the lower end of the income distribution in the United States means that the gap in disposable incomes between the bottom and the middle will be larger in the United States, which also works to offset the effects of smaller differences in teen parenthood, single parenthood, and early education.

These patterns are, of course, entirely descriptive, and the factors we were able to consider are only a subset of those that contribute to gaps in test scores and that may be associated differently with income across countries. Differences in the costs associated with high-quality health care and childcare will affect the extent to which lower-income families are able to access similar provision to their higher-income counterparts, with consequent implications for disparities in child development. We might expect the more extensive use of private markets for both these services in the United States (in contrast, for example, to the universal free health care and preschool available in the United Kingdom) to result in larger gaps in quality of provision between income groups. The existence of highly targeted programs such as Medicaid and Head Start in the United States may lead to smaller disparities between the bottom and the middle than between the middle and the top, mirroring the pattern in gaps in test scores presented in this article. The racial composition of the United States and the historical inequities with which it is associated do not have a direct counterpart in the other countries, and these factors may lead to further differentiation of income groups in the United States. The extent to which residential segregation and inequalities in neighborhood conditions differ across countries and the contribution of this to test score gaps are additional aspects on which further research is needed. The finding of the smaller test score gap between the bottom- and middle-income groups in Canada than the United States is particularly intriguing, and we have shown that the Q1–Q3 gaps in disposable incomes and in the proportion of parents born in another country are the smallest of all four countries in Canada and the largest in the United States. However, the reasons for Canada’s success in limiting the consequences of early deprivation merit further investigation.

The findings presented here demonstrate that a given degree of income inequality is consistent with quite different gaps in early childhood development, and we have documented some of the ways in which it is also consistent with different distributions of the nonmonetary resources available to children across countries. That income is transmitted across generations is indisputable. Our work suggests that to understand the consequences of the parental income distribution for the next generation, we must consider it in the context of the society that generates it and in which children grow up. Our cross-national comparison adds to evidence that trends toward an increasing concentration of financial resources among parents who are most advantaged in other ways (as in the United States) are likely to lead to more divergence in children’s destinies, even if income inequality itself remains unchanged.

## Electronic supplementary material

ESM 1 (PDF 370 kb)

## Footnotes

1 In a more recent U.S. cohort study—the ECLS Birth Cohort—test developers collapsed the separate measures of children’s vocabulary and literacy into a single scale, explaining that “it was determined that separate language and literacy scores were no longer appropriate” (Najarian et al. 2010:76–77). In addition, Bradbury et al. (2015b) showed that the magnitude of the cross-national differences in test score gaps in the measures used here are extremely robust to plausible variation in the reliability of the different test scores.

2 Disposable income is arguably a more appropriate concept than pretax income when thinking about the material resources available to children and families. Unfortunately, the information required to calculate individual family income tax and social insurance payments is not available in all the surveys. We discuss how differences in tax rates across countries may affect our conclusions in a later section.

3 Specifically, we take nationally representative household data from LIS from the following years, which correspond to years when our cohort children were between ages 6 and 9: United States 2000, United Kingdom 2010, Australia 2008, and Canada 2000. For comparability with the income group definitions used in our survey data, we weight households by the number of children under 13, convert to 2011 U.S. dollars, equivalize by 2 / √*N*, and divide each sample into five groups, imposing the U.S. gross income quintile boundaries (as calculated in the LIS data) across all the countries. Although the income quintile groups calculated using LIS are not strictly comparable with those constructed from the cohort data, these data are sufficient to show how tax rates vary across the income distribution in the different countries. More information on the LIS study is available online (lisdatacenter.org).

## References

[CR1] Almond D, Currie J, Ashenfelter O, Card D (2011). Human capital development before age five. Handbook of labor economics.

[CR2] Australian Institute of Family Studies (AIFS) (2013). Longitudinal study of Australian children: Data user guide—August 2013.

[CR3] Bailey MJ, Dynarski SM (2011). Gains and gaps: Changing inequality in U.S. college entry and completion (NBER Working Paper No. 17633).

[CR4] Berger LM, Paxson C, Waldfogel J (2009). Income and child development. Children and Youth Services Review.

[CR5] Björklund A, Jäntti M, Salverda W, Nolan B, Smeeding TM (2009). Intergenerational income mobility and the role of family background. The Oxford handbook of economic inequality.

[CR6] Black S, Devereux P, Ashenfelter O, Card D (2011). Recent developments in intergenerational mobility. Handbook of labor economics.

[CR7] Blanden J, Goodman A, Gregg P, Machin S, Corak M (2004). Changes in intergenerational mobility in Britain. Generational income mobility in North America and Europe.

[CR8] Blanden, J., Gregg, P., & Macmillan, L. (2007). Accounting for intergenerational income persistence: Noncognitive skills. ability and education, *Economic Journal, 117,* C43–C60.

[CR9] Blanden J, Haveman R, Smeeding T, Wilson K (2014). Intergenerational mobility in the United States and Great Britain: A comparative study of parent–child pathways. Review of Income and Wealth.

[CR10] Blanden J, Machin S, Hansen K, Joshi H, Dex S (2010). Intergenerational inequality in early years assessments. Children of the 21st century.

[CR11] Bradbury B, Corak M, Waldfogel J, Washbrook E, Ermisch J, Jäntti M, Smeeding TM (2012). Inequality in early child outcomes. From parents to children: The intergenerational transmission of advantage.

[CR12] Bradbury, B., Corak, M., Waldfogel, J., & Washbrook, E. (2015a). *Too many children left behind: The U.S. achievement gap in comparative perspective.* New York, NY: Russell Sage Foundation.

[CR13] Bradbury, B., Corak, M., Waldfogel, J., & Washbrook, E. (2015b).* Too many children left behind: The U.S. achievement gap in comparative perspective* (Technical appendix). Available from: https://www.russellsage.org/publications/too-many-children-left-behind

[CR14] Bradley RH, Corwyn RF (2002). Socioeconomic status and child development. Annual Review of Psychology.

[CR15] Bronfenbrenner U, Postlethwaite TN, Husen T (1994). Ecological models of human development. International encyclopedia of education.

[CR16] Carneiro P, Heckman JJ, Heckman J, Krueger A (2003). Human capital policy. Inequality in America: What role for human capital policy?.

[CR17] Carneiro P, Meghir C, Parey M (2013). Maternal education, home environments, and the development of children and adolescents. Journal of the European Economic Association.

[CR18] Case A, Lubotsky D, Paxson C (2002). Economic status and health in childhood: The origins of the gradient. American Economic Review.

[CR19] Chetty R, Hendren N, Kline P, Saez E (2014). Where is the land of opportunity? The geography of intergenerational mobility in the United States. Quarterly Journal of Economics.

[CR20] Conger RD, Conger KJ, Elder GH, Lorenz FO, Simons RL, Whitbeck LB (1992). A family process model of economic hardship and adjustment of early adolescent boys. Child Development.

[CR21] Conger RD, McCarty JA, Yang RK, Lahey BB, Burgess RL (1984). Mother’s age as a predictor of observed maternal behavior in three independent samples of families. Journal of Marriage and the Family.

[CR22] Corak M, Creedy J, Kalb G (2006). Do poor children become poor adults? Lessons from a cross-country comparison of generational earnings mobility. Dynamics of inequality and poverty: Research on economic inequality.

[CR23] Corak M (2013). Income inequality, equality of opportunity, and intergenerational mobility. Journal of Economic Perspectives.

[CR24] Crosnoe R, Turley RNL (2011). K–12 educational outcomes of immigrant youth. Future of Children.

[CR25] Cunha F, Heckman JJ, Lochner L, Masterov DV, Hanushek E, Welch F (2006). Interpreting the evidence on life cycle skill formation. Handbook of the economics of education.

[CR26] Duncan, G. J., & Brooks-Gunn, J. (Eds.) (1997). *Consequences of growing up poor*. New York, NY: Russell Sage Foundation.

[CR27] Duncan GJ, Magnuson K (2013). Investing in preschool programs. Journal of Economic Perspectives.

[CR28] Dunn, L, M., & Dunn, L. M. (1981). *PPVT-R manual* (Rev. ed.). Circle Pines, MN: American Guidance Service.

[CR29] Dunn, L. M., Dunn, L. M., & Thériault-Whalen, C. M. (1993). *Échelle de vocabulaire en images Peabody: Adaptation Française du peabody picture vocabulary test-revised* [Vocabulary scale in Peabody pictures: French adaptation of Peabody Picture Vocabulary Test-Revised]. Toronto, Canada: PsyCan.

[CR30] Dunn, L. M., Dunn, L. M., & Dunn, D. M. (1997). *The Peabody Picture Vocabulary Test *(3rd ed.). Circle Pines, MN: American Guidance Service Inc. Publishing.

[CR31] Elliott, C. D., Smith, P., & McCulloch, K. (1997). *British Ability Scales second edition (BAS II): Technical manual*. London, UK: NFER-Nelson.

[CR32] Ermisch JF, Francesconi M (2001). Family structure and children’s achievements. Journal of Population Economics.

[CR33] Ermisch J, Jäntti M, Smeeding TM (2012). From parents to children: The intergenerational transmission of advantage.

[CR34] Ermisch J, Jäntti M, Smeeding T, Watson J, Ermish J, Jäntti M, Smeeding TM (2012). Advantage in comparative perspective. From parents to children: The intergenerational transmission of advantage.

[CR35] Fletcher JM, Wolfe B (2016). The importance of family income in the formation and evolution of non-cognitive skills in childhood. Economics of Education Review.

[CR36] Geronimus AT, Korenman S, Hillemeier MM (1994). Does young maternal age adversely affect child development? Evidence from cousin comparisons in the United States. Population and Development Review.

[CR37] Guo G, Harris KM (2000). The mechanisms mediating the effects of poverty on children’s intellectual development. Demography.

[CR38] Hansen K (2014). Millennium Cohort Study: A guide to the datasets.

[CR39] Jäntti, M., Bratsberg, B., Røed, K., Raaum, O., Naylor, R., Österbacka, E., . . . Eriksson, T. (2006). *American exceptionalism in a new light: A comparison of intergenerational earnings mobility in the Nordic countries, the United Kingdom and the United States* (IZA Discussion Paper No. 1938). Bonn, Germany: Institute for the Study of Labor.

[CR40] Knudsen EI, Heckman JJ, Cameron JL, Shonkoff JP (2006). Economic, neurobiological, and behavioral perspectives on building America’s future workforce. Proceedings of the National Academy of Sciences.

[CR41] Lee CI, Solon G (2009). Trends in intergenerational income mobility. Review of Economics and Statistics.

[CR42] Linver MR, Brooks-Gunn J, Kohen DE (2002). Family processes as pathways from income to young children’s development. Developmental Psychology.

[CR43] Lucas-Thompson RG, Goldberg WA, Prause J (2010). Maternal work early in the lives of children and its distal associations with achievement and behavior problems: A meta-analysis. Psychological Bulletin.

[CR44] Magnuson K (2007). Maternal education and children’s academic achievement during middle childhood. Developmental Psychology.

[CR45] Magnuson K, Waldfogel J, Washbrook E, Ermisch J, Jäntti M, Smeeding TM (2012). SES gradients in skills during the school years. From parents to children: The intergenerational transmission of advantage.

[CR46] Martinson, M. L., & Reichman, N. E. (2016). Socioeconomic inequalities in low birth weight in the United States, the United Kingdom, Canada, and Australia. *American Journal of Public Health, 106,* 748–754. 10.2105/AJPH.2015.303007 PMC4986052 26794171

[CR47] Mazumder B, Bowles S, Gintis H, Groves MO (2005). The apple falls even closer to the tree than we thought: New and revised estimates of the intergenerational inheritance of earnings. Unequal chances: Family background and economic success.

[CR48] McLanahan S (2004). Diverging destinies: How children are faring under the second demographic transition. Demography.

[CR49] McLanahan S, Jacobsen W, Amato PR, McHale SL, Booth A, Hook J (2015). Diverging destinies revisited. Diverging destinies: Families in an era of increasing inequality.

[CR50] McLanahan S, Sandefur G (1994). Growing up with a single parent: What hurts, what helps.

[CR51] McLoyd VC (1998). Socioeconomic disadvantage and child development. American Psychologist.

[CR52] Najarian, M., Snow, K., Lennon, J., & Kinsey, S. (2010). *Early Childhood Longitudinal Study, Birth Cohort (ECLS-B), Preschool–kindergarten 2007 psychometric report *(NCES 2010-009). Washington, DC: National Center for Education Statistics, Institute of Education Sciences, U.S. Department of Education.

[CR53] Nolan B, Esping-Anderson G, Whelan CT, Maitre B, Wagner S, Smeeding TM, Erikson R, Jäntti M (2011). The role of social institutions in intergenerational mobility. Persistence, privilege and parenting: The comparative study of intergenerational mobility.

[CR54] Organisation for Economic Co-operation and Development (OECD) (2013). PISA 2012 results: Excellence through equity: Giving every student the chance to succeed.

[CR55] Reardon SF, Duncan GJ, Murnane RJ (2011). The widening academic-achievement gap between the rich and the poor: New evidence and possible explanations. Whither opportunity? Rising inequality, schools, and children’s life chances.

[CR56] Rothman, S. (2005). *Report on adapted PPVT-III and Who Am I? *(Growing Up in Australia: The Longitudinal Study of Australian Children; Data Issues Paper No. 2). Melbourne, AU: Australian Institute of Family Studies, Australian Council for Educational Research.

[CR57] Rothstein J, Wozny N (2013). Permanent income and the black-white test score gap. Journal of Human Resources.

[CR58] Solon, G. (1999). Intergenerational mobility in the labour market. In O. Ashenfelter & D. Card (Eds.), *Handbook of labor economics* (Vol. 3A, pp. 1761–1800). Amsterdam, the Netherlands: North Holland.

[CR59] Solon G (2002). Cross-country differences in intergenerational earnings mobility. Journal of Economic Perspectives.

[CR60] Solon G, Corak M (2004). A model of intergenerational mobility variation over time and place. Generational income mobility in North America and Europe.

[CR61] Statistics Canada. (2007). *Microdata user guide: National Longitudinal Survey of Children and Youth, Cycle 8, September 2008 to July 2009*. Retrieved from http://www23.statcan.gc.ca/imdb-bmdi/document/4450_D4_T9_V8-eng.pdf

[CR62] Tourangeau, K., Nord, C., Le, T., Sorongon, A. G., Najarian, M., & Hausken, E. G. (2009). *Early Childhood Longitudinal Study, kindergarten class of 1998–99 (ECLS-K): Combined user’s manual for the ECLS-K eighth-grade and K–8 full sample data files and electronic codebooks *(NCES No. 2009-004). Washington, DC: National Center for Education Statistics, Institute of Education Sciences, U.S. Department of Education.

[CR63] Wadsworth ME, Ahlkvist JA, Amato PR, McHale SL, Booth A, Hook J (2015). Inequality begins outside the home: Putting parental educational investments into context. Diverging destinies: Families in an era of increasing inequality.

[CR64] Waldfogel J, Han WJ, Brooks-Gunn J (2002). The effects of early maternal employment on child cognitive development. Demography.

[CR65] Waldfogel J, Washbrook E, Smeeding T, Erikson R, Jäntti M (2011). Income-related gaps in school readiness in the United States and the United Kingdom. Persistence, privilege and parenting: The comparative study of intergenerational mobility.

[CR66] Washbrook E, Gregg P, Propper C (2014). A decomposition analysis of the relationship between parental income and multiple child outcomes. Journal of the Royal Statistical Society: Series A (Statistics in Society).

[CR67] Washbrook E, Waldfogel J, Corak M, Bradbury B, Ghanghro A (2012). The development of young children of immigrants in Australia, Canada, the United Kingdom, and the United States. Child Development..

[CR68] Yoshikawa, H., Weiland, C., Brooks-Gunn, J., Burchinal, M. R., Espinosa, L. M., Gormley, W. T., . . . Zaslow, M. J. (2013). *Investing in our future: The evidence base on preschool education*. New York, NY: Foundation for Child Development; and Ann Arbor, MI: Society for Research in Child Development.

