# Supplementary material for: Income-Related Gaps in Early Child Cognitive Development: Why Are They Larger in the United States Than in the United Kingdom, Australia, and Canada?
Source: Demography. 2018 Nov 28;56(1):367–90. doi: 10.1007/s13524-018-0738-8 (PMC6513808; doi:10.1007/s13524-018-0738-8)
Supplement: Supplementary file 1 — (PDF 370 kb) [file 13524_2018_738_MOESM1_ESM.pdf]

ONLINE DATA APPENDIX to Income-Related Gaps in Early Child Cognitive Development: Why Are They Larger in the United States Than in the United Kingdom, Australia, and Canada?

Table OA1. Sample sizes

	Income group				
	Q1	Q2	Q3	Q4	Q5
US	1679	1680	1663	1675	1673
UK	2443	2371	2641	2794	1513
AU	712	945	1053	800	389
CA	445	946	1308	1087	512

Table OA2. Sample estimates

	Means by income group					Gaps within countries (ref = Q3) $[D_{c,q}]$				Difference in gaps between US and other countries $[DD_{c,q}]$				Overall summary	
														Gap w/in country	US diff in gap
	Q1	Q2	Q3	Q4	Q5	Q1-Q3	Q2-Q3	Q4-Q3	Q5-Q3	Q1-Q3	Q2-Q3	Q4-Q3	Q5-Q3	Q5-Q1	Q5-Q1
**Standardized early literacy test scores**															
US	-0.660	-0.345	-0.012	0.363	0.596	-0.648	-0.333	0.375	0.608	-	-	-	-	1.256	-
	(0.055)	(0.042)	(0.040)	(0.041)	(0.050)	(0.068)**	(0.058)**	(0.057)**	(0.064)**					(0.074)**	
UK	-0.586	-0.179	0.104	0.284	0.501	-0.690	-0.282	0.180	0.398	0.042	-0.051	0.194	0.210	1.087	0.169
	(0.047)	(0.030)	(0.023)	(0.023)	(0.035)	(0.052)**	(0.037)**	(0.032)**	(0.042)**	(0.086)	(0.069)	(0.066)**	(0.076)**	(0.059)**	(0.095)+
AU	-0.384	-0.133	0.081	0.227	0.320	-0.465	-0.214	0.145	0.239	-0.183	-0.119	0.229	0.369	0.704	0.551
	(0.056)	(0.040)	(0.031)	(0.038)	(0.048)	(0.065)**	(0.051)**	(0.049)**	(0.057)**	(0.094)+	(0.077)	(0.076)**	(0.086)**	(0.074)**	(0.105)**
CA	-0.307	-0.229	-0.033	0.185	0.316	-0.274	-0.196	0.218	0.350	-0.374	-0.137	0.157	0.258	0.623	0.632
	(0.065)	(0.054)	(0.059)	(0.047)	(0.084)	(0.088)**	(0.080)*	(0.076)**	(0.103)**	(0.111)**	(0.099)	(0.095)+	(0.121)*	(0.106)**	(0.130)**

	Means by income group					Gaps within countries (ref = Q3) $[D_{c,q}]$				Difference in gaps between US and other countries $[DD_{c,q}]$				Overall summary: Gap w/in country	Overall summary: US diff in gap
	Q1	Q2	Q3	Q4	Q5	Q1-Q3	Q2-Q3	Q4-Q3	Q5-Q3	Q1-Q3	Q2-Q3	Q4-Q3	Q5-Q3	Q5-Q1	Q5-Q1
**Mothers with college degree (proportion)**															
US	0.014	0.061	0.132	0.298	0.609	-0.119	-0.071	0.166	0.477	-	-	-	-	0.595	-
	(0.004)	(0.009)	(0.014)	(0.018)	(0.019)	(0.014)**	(0.016)**	(0.022)**	(0.023)**					(0.019)**	
UK	0.005	0.030	0.104	0.269	0.569	-0.099	-0.074	0.165	0.465	-0.019	0.003	0.001	0.012	0.564	0.031
	(0.001)	(0.004)	(0.007)	(0.014)	(0.021)	(0.007)**	(0.008)**	(0.016)**	(0.022)**	(0.016)	(0.018)	(0.027)	(0.032)	(0.021)**	(0.028)
AU	0.089	0.140	0.218	0.376	0.633	-0.129	-0.078	0.159	0.415	0.010	0.006	0.007	0.062	0.544	0.052
	(0.011)	(0.011)	(0.012)	(0.017)	(0.023)	(0.016)**	(0.016)**	(0.020)**	(0.025)**	(0.022)	(0.023)	(0.030)	(0.034)+	(0.025)**	(0.032)
CA	0.031	0.065	0.117	0.250	0.535	-0.086	-0.052	0.133	0.417	-0.033	-0.019	0.032	0.059	0.503	0.092
	(0.012)	(0.015)	(0.014)	(0.023)	(0.036)	(0.018)**	(0.021)*	(0.026)**	(0.038)**	(0.023)	(0.026)	(0.034)	(0.045)	(0.038)**	(0.042)*
**Mothers with a high school diploma or less (proportion)**															
US	0.784	0.602	0.459	0.272	0.123	0.325	0.142	-0.187	-0.336	-	-	-	-	-0.661	-
	(0.023)	(0.024)	(0.021)	(0.019)	(0.013)	(0.031)**	(0.032)**	(0.029)**	(0.025)**					(0.026)**	
UK	0.868	0.688	0.505	0.298	0.118	0.363	0.183	-0.207	-0.387	-0.038	-0.041	0.020	0.051	-0.749	0.088
	(0.009)	(0.012)	(0.012)	(0.014)	(0.010)	(0.015)**	(0.017)**	(0.019)**	(0.016)**	(0.034)	(0.036)	(0.034)	(0.030)+	(0.013)**	(0.030)**
AU	0.792	0.691	0.587	0.434	0.195	0.205	0.104	-0.153	-0.392	0.120	0.039	-0.034	0.056	-0.597	-0.065
	(0.016)	(0.016)	(0.015)	(0.017)	(0.020)	(0.022)**	(0.021)**	(0.022)**	(0.025)**	(0.038)**	(0.039)	(0.036)	(0.035)	(0.026)**	(0.037)+
CA	0.635	0.438	0.328	0.171	0.098	0.307	0.111	-0.156	-0.230	0.018	0.032	-0.031	-0.106	-0.537	-0.124
	(0.038)	(0.027)	(0.024)	(0.019)	(0.023)	(0.045)**	(0.036)**	(0.031)**	(0.033)**	(0.054)	(0.048)	(0.042)	(0.042)*	(0.044)**	(0.051)*
**Two resident biological parents at child's age 5 (proportion)**															
US	0.382	0.546	0.656	0.798	0.862	-0.274	-0.110	0.142	0.206	-	-	-	-	0.480	-
	(0.026)	(0.027)	(0.024)	(0.018)	(0.018)	(0.036)**	(0.036)**	(0.030)**	(0.030)**					(0.031)**	
UK	0.369	0.637	0.846	0.941	0.980	-0.477	-0.209	0.095	0.134	0.203	0.099	0.046	0.072	0.610	-0.130
	(0.026)	(0.018)	(0.016)	(0.012)	(0.010)	(0.031)**	(0.024)**	(0.020)**	(0.019)**	(0.047)**	(0.043)*	(0.036)	(0.036)*	(0.028)**	(0.042)**
AU	0.507	0.808	0.917	0.954	0.968	-0.409	-0.109	0.037	0.051	0.135	-0.002	0.105	0.155	0.461	0.019
	(0.025)	(0.019)	(0.015)	(0.015)	(0.013)	(0.029)**	(0.024)**	(0.021)+	(0.020)*	(0.046)**	(0.044)	(0.037)**	(0.036)**	(0.028)**	(0.042)
CA	0.421	0.723	0.857	0.909	0.939	-0.436	-0.134	0.052	0.083	0.162	0.023	0.090	0.123	0.518	-0.038
	(0.044)	(0.028)	(0.027)	(0.022)	(0.022)	(0.052)**	(0.039)**	(0.035)	(0.035)*	(0.063)*	(0.054)	(0.046)+	(0.046)**	(0.049)**	(0.058)

	Means by income group					Gaps within countries (ref = Q3) $[D_{c,q}]$				Difference in gaps between US and other countries $[DD_{c,q}]$				Overall summary	
														Gap w/in country	US diff in gap
	Q1	Q2	Q3	Q4	Q5	Q1-Q3	Q2-Q3	Q4-Q3	Q5-Q3	Q1-Q3	Q2-Q3	Q4-Q3	Q5-Q3	Q5-Q1	Q5-Q1
**Mothers under age 20 at birth of child (proportion)**															
US	0.226	0.183	0.150	0.052	0.009	0.077	0.033	-0.097	-0.141	-	-	-	-	-0.217	-
	(0.019)	(0.017)	(0.015)	(0.008)	(0.002)	(0.024)**	(0.022)	(0.017)**	(0.015)**					(0.019)**	
UK	0.201	0.072	0.028	0.008	0.000	0.174	0.044	-0.020	-0.028	-0.097	-0.011	-0.077	-0.113	-0.201	-0.016
	(0.014)	(0.007)	(0.005)	(0.002)	(0.000)	(0.015)**	(0.009)**	(0.005)**	(0.005)**	(0.028)**	(0.024)	(0.018)**	(0.016)**	(0.014)**	(0.023)
AU	0.081	0.041	0.017	0.009	0.000	0.064	0.024	-0.008	-0.017	0.013	0.009	-0.089	-0.124	-0.081	-0.136
	(0.014)	(0.009)	(0.004)	(0.005)	(0.000)	(0.014)**	(0.010)*	(0.007)	(0.004)**	(0.028)	(0.024)	(0.018)**	(0.015)**	(0.014)**	(0.023)**
CA	-	-	-	-	-	-	-	-	-	-	-	-	-	-	-
**Immigrant parent (proportion)**															
US	0.312	0.266	0.167	0.102	0.133	0.145	0.099	-0.065	-0.034	-	-	-	-	-0.179	-
	(0.031)	(0.018)	(0.015)	(0.010)	(0.012)	(0.034)**	(0.024)**	(0.018)**	(0.019)+					(0.033)**	
UK	0.240	0.147	0.117	0.118	0.163	0.122	0.030	0.001	0.045	0.023	0.069	-0.066	-0.079	-0.077	-0.102
	(0.028)	(0.012)	(0.011)	(0.009)	(0.014)	(0.031)**	(0.016)+	(0.014)	(0.018)*	(0.046)	(0.029)*	(0.023)**	(0.026)**	(0.032)*	(0.045)*
AU	0.370	0.342	0.310	0.353	0.406	0.059	0.032	0.043	0.096	0.086	0.067	-0.108	-0.130	0.036	-0.216
	(0.025)	(0.018)	(0.015)	(0.018)	(0.020)	(0.029)*	(0.023)	(0.024)+	(0.025)**	(0.045)+	(0.033)*	(0.030)**	(0.031)**	(0.032)	(0.046)**
CA	0.252	0.218	0.214	0.221	0.233	0.039	0.004	0.007	0.020	0.107	0.095	-0.072	-0.054	-0.019	-0.160
	(0.047)	(0.028)	(0.024)	(0.025)	(0.037)	(0.053)	(0.037)	(0.035)	(0.044)	(0.063)+	(0.044)*	(0.040)+	(0.048)	(0.059)	(0.068)*

	Means by income group					Gaps within countries (ref = Q3) $[D_{c,q}]$				Difference in gaps between US and other countries $[DD_{c,q}]$				Overall summary: Gap w/in country	Overall summary: US diff in gap
	Q1	Q2	Q3	Q4	Q5	Q1-Q3	Q2-Q3	Q4-Q3	Q5-Q3	Q1-Q3	Q2-Q3	Q4-Q3	Q5-Q3	Q5-Q1	Q5-Q1
**Average maternal weekly work hours at child's age 5 (all)**															
US	18.128	24.622	27.193	27.755	25.226	-9.065	-2.571	0.561	-1.967	-	-	-	-	7.098	-
	(0.839)	(0.774)	(0.759)	(0.803)	(0.723)	(1.131)**	(1.084)*	(1.105)	(1.048)+					(1.107)**	
UK	3.196	10.645	16.539	19.010	22.338	-13.343	-5.895	2.471	5.799	4.278	3.323	-1.910	-7.766	19.142	-12.043
	(0.301)	(0.435)	(0.393)	(0.387)	(0.665)	(0.495)**	(0.586)**	(0.551)**	(0.772)**	(1.235)**	(1.232)**	(1.235)	(1.302)**	(0.730)**	(1.326)**
AU	5.876	10.481	15.658	17.759	21.016	-9.782	-5.177	2.101	5.358	0.717	2.606	-1.539	-7.325	15.140	-8.042
	(0.680)	(0.596)	(0.507)	(0.598)	(0.778)	(0.848)**	(0.782)**	(0.784)**	(0.929)**	(1.414)	(1.337)+	(1.355)	(1.400)**	(1.034)**	(1.515)**
CA	12.093	17.884	22.872	29.316	28.818	-10.779	-4.988	6.444	5.945	1.714	2.417	-5.883	-7.912	16.725	-9.627
	(1.318)	(1.004)	(0.905)	(0.708)	(1.444)	(1.599)**	(1.352)**	(1.149)**	(1.704)**	(1.958)	(1.733)	(1.594)**	(2.001)**	(1.955)**	(2.246)**
**Mothers with a paid job at child's age 5 (proportion)**															
US	0.496	0.678	0.738	0.779	0.713	-0.242	-0.060	0.041	-0.025	-	-	-	-	0.217	-
	(0.028)	(0.019)	(0.018)	(0.018)	(0.018)	(0.034)**	(0.026)*	(0.025)	(0.025)					(0.034)**	
UK	0.161	0.444	0.687	0.760	0.769	-0.526	-0.243	0.073	0.082	0.284	0.183	-0.033	-0.107	0.608	-0.391
	(0.010)	(0.012)	(0.013)	(0.011)	(0.018)	(0.017)**	(0.018)**	(0.017)**	(0.022)**	(0.038)**	(0.031)**	(0.031)	(0.034)**	(0.021)**	(0.039)**
AU	0.270	0.473	0.658	0.706	0.757	-0.388	-0.185	0.048	0.099	0.146	0.125	-0.008	-0.124	0.487	-0.270
	(0.017)	(0.017)	(0.014)	(0.015)	(0.019)	(0.022)**	(0.022)**	(0.021)*	(0.024)**	(0.040)**	(0.034)**	(0.033)	(0.035)**	(0.025)**	(0.042)**
CA	0.369	0.600	0.738	0.881	0.850	-0.370	-0.138	0.142	0.112	0.127	0.078	-0.102	-0.137	0.481	-0.264
	(0.039)	(0.026)	(0.022)	(0.015)	(0.035)	(0.044)**	(0.034)**	(0.026)**	(0.041)**	(0.056)*	(0.043)+	(0.037)**	(0.048)**	(0.052)**	(0.062)**
**Average maternal weekly work hours at child's age 5 (employed only)**															
US	36.563	36.319	36.833	35.636	35.377	-0.270	-0.514	-1.198	-1.456	-	-	-	-	-1.186	-
	(0.583)	(0.644)	(0.586)	(0.646)	(0.574)	(0.827)	(0.871)	(0.872)	(0.820)+					(0.818)	
UK	19.857	23.969	24.073	25.000	29.040	-4.215	-0.104	0.927	4.967	3.945	-0.410	-2.125	-6.423	9.182	-10.368
	(0.722)	(0.587)	(0.355)	(0.344)	(0.554)	(0.805)**	(0.686)	(0.494)+	(0.658)**	(1.154)**	(1.109)	(1.002)*	(1.052)**	(0.910)**	(1.224)**
AU	21.798	22.162	23.802	25.149	27.770	-2.004	-1.640	1.348	3.969	1.734	1.126	-2.545	-5.425	5.973	-7.159
	(1.258)	(0.785)	(0.525)	(0.600)	(0.724)	(1.363)	(0.944)+	(0.797)+	(0.894)**	(1.594)	(1.284)	(1.182)*	(1.214)**	(1.451)**	(1.666)**
CA	32.814	29.803	30.984	33.290	33.913	1.830	-1.180	2.306	2.930	-2.101	0.667	-3.504	-4.386	1.099	-2.285
	(1.760)	(1.031)	(0.813)	(0.563)	(1.013)	(1.939)	(1.313)	(0.989)*	(1.299)*	(2.108)	(1.576)	(1.319)**	(1.537)**	(2.031)	(2.190)

	Means by income group					Gaps within countries (ref = Q3) $[D_{c,q}]$				Difference in gaps between US and other countries $[DD_{c,q}]$				Overall summary: Gap w/in country	Overall summary: US diff in gap
	Q1	Q2	Q3	Q4	Q5	Q1-Q3	Q2-Q3	Q4-Q3	Q5-Q3	Q1-Q3	Q2-Q3	Q4-Q3	Q5-Q3	Q5-Q1	Q5-Q1
**Children in center-based care before school entry (proportion)**															
US	0.624	0.645	0.621	0.712	0.829	0.003	0.024	0.091	0.208	-	-	-	-	0.205	-
	(0.039)	(0.033)	(0.025)	(0.031)	(0.027)	(0.046)	(0.041)	(0.040)*	(0.037)**					(0.047)**	
UK	0.906	0.932	0.933	0.941	0.921	-0.027	-0.001	0.008	-0.012	0.030	0.025	0.083	0.220	0.015	0.190
	(0.029)	(0.023)	(0.017)	(0.016)	(0.021)	(0.033)	(0.029)	(0.023)	(0.027)	(0.057)	(0.050)	(0.046)+	(0.046)**	(0.036)	(0.059)**
AU	0.883	0.944	0.954	0.977	0.979	-0.071	-0.010	0.023	0.024	0.074	0.034	0.068	0.184	0.096	0.110
	(0.028)	(0.019)	(0.018)	(0.022)	(0.033)	(0.033)*	(0.026)	(0.028)	(0.038)	(0.057)	(0.048)	(0.049)	(0.053)**	(0.043)*	(0.064)+
CA	0.399	0.483	0.596	0.625	0.695	-0.197	-0.113	0.029	0.099	0.200	0.137	0.062	0.109	0.296	-0.091
	(0.054)	(0.045)	(0.043)	(0.047)	(0.069)	(0.069)**	(0.062)+	(0.064)	(0.082)	(0.083)*	(0.075)+	(0.075)	(0.090)	(0.088)**	(0.100)

Notes: Standard errors in parentheses. + significant at 10% level, * significant at 5% level, ** at 1% level. Estimates weighted using longitudinal survey weights. Canadian numbers for teenage motherhood suppressed due to small cell sizes.
